# Epigenetic Regulation of Inflammatory Cytokines and Associated Genes in Human Malignancies

**DOI:** 10.1155/2015/201703

**Published:** 2015-03-01

**Authors:** Rehana Yasmin, Sami Siraj, Amjad Hassan, Abdul Rehman Khan, Rashda Abbasi, Nafees Ahmad

**Affiliations:** ^1^Department of Environmental Sciences, COMSATS Institute of Information Technology, Abbottabad 22060, Pakistan; ^2^Institute of Basic Medical Sciences, Khyber Medical University, Peshawar 25124, Pakistan; ^3^Institute of Biomedical and Genetic Engineering, Islamabad 54000, Pakistan

## Abstract

Inflammation is a multifaceted defense response of immune system against infection. Chronic inflammation has been implicated as an imminent threat for major human malignancies and is directly linked to various steps involved in tumorigenesis. Inflammatory cytokines, interleukins, interferons, transforming growth factors, chemokines, and adhesion molecules have been associated with chronic inflammation. Numerous cytokines are reported to be aberrantly regulated by different epigenetic mechanisms like DNA methylation and histone modifications in tumor tissues, contributing to pathogenesis of tumor in multiple ways. Some of these cytokines also work as epigenetic regulators of other crucial genes in tumor biology, either directly or indirectly. Such regulations are reported in lung, breast, cervical, gastric, colorectal, pancreatic, prostate, and head and neck cancers. Epigenetics of inflammatory mediators in cancer is currently subject of extensive research. These investigations may help in understanding cancer biology and to develop effective therapeutic strategies. The purpose of this paper is to have a brief view of the aberrant regulation of inflammatory cytokines in human malignancies.

## 1. Introduction

Inflammation is a complex defense response of immune system, attempted to neutralize an insult and reestablish normal tissue structure and function [1]. Inflammation is characterized by redness, swelling, and pain and sometimes failure of function. It is mainly mediated and regulated by inflammatory cytokines. Proinflammatory cytokines are concerned with the upgrading of inflammatory reactions while anti-inflammatory cytokines dampen the proinflammatory cytokine response. Chronic inflammation has been associated with different diseases, such as cancer, diabetes, cardiovascular disorders, pulmonary, and neurological diseases [2–6].

Chronic inflammation is now well acknowledged as a threat feature for major types of cancer [7–11]. About 25% of all cancers are connected to chronic inflammation, which is linked to various stages of tumorigenesis including cellular transformation, tumor progression, endurance, propagation, invasion, angiogenesis, and metastasis [12–14]. Proinflammatory cytokines like chemokines, adhesion molecules, and inflammatory enzymes are known to cause chronic inflammation. Several proinflammatory genes, for example, tumor necrosis factor (TNF) and members of its superfamily, IL-1a, IL-1b, IL-6, IL-8, IL-18, chemokines, VEGF, MMP-9, 5-LOX, and COX-2, play critical role in the control of apoptosis, angiogenesis, proliferation, invasion, and metastasis. Overexpression of transcription factors like NF-*κ*B, which becomes constitutively active in most tumors, is principally responsible for the expression of these genes [15–17].

The survival and growth of the initiated cells are prerequisite for tumor development. Many inflammatory mediators such as interleukins, eicosanoids, and chemokines are able to motivate the propagation of both normal and cancer cells [18]. Clinical and epidemiological studies have suggested a strong connection between cancer, inflammation, and chronic infection [12, 19–22]. Proinflammatory mediators can contribute to tumor promotion and progression when produced intolerably and persistently because of events like aberrant epigenetic changes [12, 18, 23, 24].

Epigenetic processes are direct heritable changes in gene expression without changes in DNA sequence [25]. Epigenetic gene silencing is the transcriptional repression of specific genes throughout growth and cellular differentiation [26, 27]. The active or silent gene states are controlled by the processes of addition or removal of chemical modifications in the chromatin [28, 29]. These modifications include DNA methylation and a variety of posttranslational histone modifications (acetylation, methylation, phosphorylation, etc.). These modifications are recognized by protein complexes that decide the fate of gene expression [30]. In mammals, DNA methylation mainly involves the attachment of methyl groups (–CH_3_) to cytosine residues present at the CpG sites. Hypermethylation of promoter regions of genes is typically associated with transcriptional silencing while hypomethylation facilitates gene expression.

A number of reviews have been published on diverse aspects of cancer epigenetics, inflammation and the relationship between the chronic inflammation and cancer. The current review is focused on epigenetic regulation of different inflammatory cytokines involved in various human malignances with emphasis on aberrant methylation.

## 2. Basic Concept of Histone Modifications and DNA Methylation

Epigenetic regulation is the mechanism by which chromatin undergoes multiple types of alterations including histone modifications and DNA methylation. These modifications are important for chromatin remodeling and accessibility of transcriptional machinery to facilitate and regulate the transfer of genetic information.

Histone modifications (like acetylation and methylation, etc.) are important regulators of gene transcription. These can activate transcription by acetylation and are depending on the level of histone methylation and repress or inactivate transcription [31]. These modifications have not only the ability to regulate the binding of effector molecules essential to DNA processes including transcription, repair, and replication, but also the ability to regulate higher order chromatin structure and stability [32]. Therefore, it is not surprising that many chromatin-modifying enzymes are deranged during malignant transformation.

**Figure 1:**
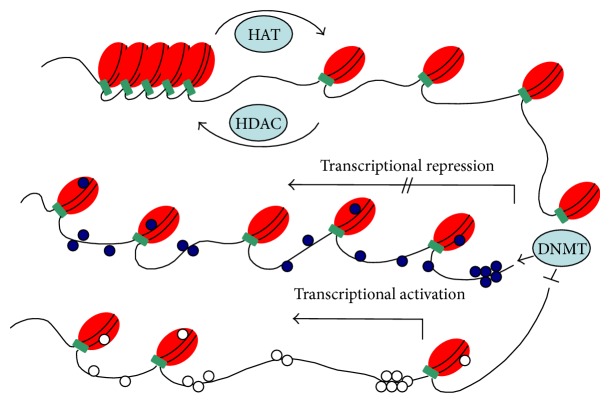
Overview of different epigenetic mechanisms involved in the regulation of chromatin (heterochromatin and euchromatin). Histone acetylation catalyzed by histone acetylase (HAT) that slightly unwraps the DNA from histones and promotes gene expression. Histone deacetylation is catalyzed by histone deacetylase (HADC), which tightens the DNA and histone core together and represses transcription. CpG islands are mostly located in the regulatory regions of genome. DNA methyltransferase (DNMT) adds methyl group to the CpG site, which is the main epigenetic mechanism reported for transcriptional repression.

Histone acetylation of the lysine residues is regulated by enzymes that catalyze the addition or removal of acetyl group at numerous positions in histones. Histone acetyltransferases (HATs) and histone deacetyltransferases (HDATs) are the enzymes that acetylate or deacetylate histones, respectively, and thus activate or suppress the transcriptional activity [33]. However, exact mechanism of this gene regulation by histone acetylation is not well understood. It is believed that acetylation process neutralizes positively charged histones and reduces their interaction with the negatively charged DNA thus loosening the chromatin structure (Figure 1). Histone methyltransferases (HMTs) are the most recently discovered histone-modifying enzymes that regulate gene expression by histone methylation at specific sites [34]. These enzymes catalyze the transfer of methyl groups to lysine and arginine residues of histones (H3 and H4). Depending upon the number of methyl groups transferred and the histone residues involved, transcription of a gene can be suppressed or activated [35].

DNA methylation is the most widely studied mechanism of epigenetic modifications meant to regulate gene expression. It involves covalent attachment of a methyl group to the cytosine residue by the DNA methyltransferase enzymes (DNMTs) [36]. In DNA methylation, cytosine is converted to methyl-cytosine at CpG (cytosine-phosphate-guanine) site. These CpG sites can span at an area of more than 200 bp (called CpG islands), are present in about 70% of human gene promoters, and are important modulators of gene transcription (Figure 1) [37–39]. The CpG islands are methylated on the inactive female X-chromosome and in certain other conditions like tissue specific and age-related genes [40]. However, in normal cells most of the CpG islands are unmethylated. The aberrant methylation of the CpG islands especially in the promoter region of tumor suppressor genes are responsible for inducing cancer and related human diseases [41].

## 3. Cytokines as Epigenetic Regulators in Cancer

Along with being epigenetically regulated in malignancies, cytokines also affect the regulation of other genes. Few studies report certain cytokines as epigenetic regulators of other genes having role in cancer initiation and progression.

**Figure 2:**
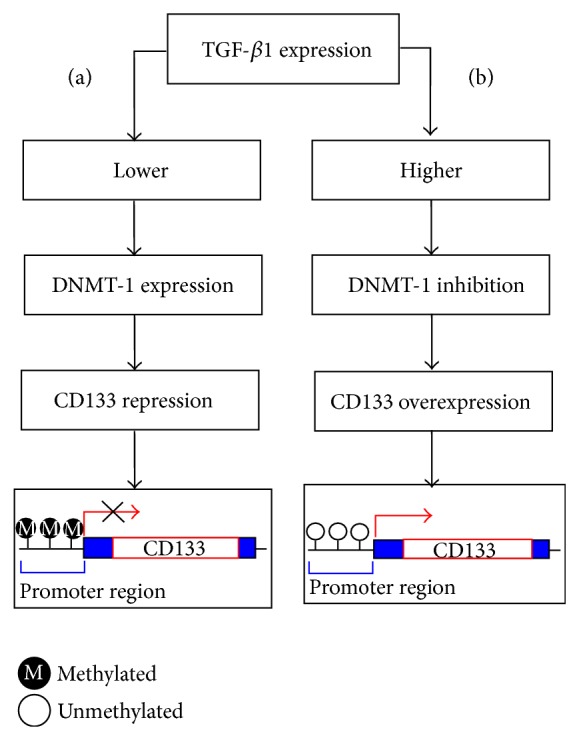
A schematic presentation elaborating epigenetic regulation of CD133 by TGF-*β*1 mediated by DNA methyltransferase (DNMT-1) in Huh 7 cell (HCC cell line). (a) Low expression of TGF-*β*1 does not affect DNMT-1 and thus methylation of CD133 promoter is maintained. (b) Elevated expression of TGF-*β*1 inhibits DNMT-1 which results in demethylation of CD133 promoter resulting in the initiation of transcription.

TGF-*β* is an inflammatory cytokine having role in organ development, cellular differentiation apoptosis, and fibrosis [42]. It regulates the expression of CD133 (gene symbol:* PROM1*) in cancer stem cells (CSCs) by DNA methylation (Figure 2) [43]. CD133 is used extensively as a stem cell marker for the identification of normal and cancer cells especially in liver [44]. The high expression of CD133 by promoter demethylation adds to the resistance of tumor cells to chemotherapy and apoptosis. TGFB1 itself and its receptors (TGFBR1 and TGFGR2) are aberrantly regulated by promoter methylation in various cancers including gastric, breast, lung, and ovarian. Downregulation of TGFB1 has also been linked with the paclitaxel resistance, a mitotic inhibitor used in cancer chemotherapy [45–51].

Suppressor of cytokine signaling 1 (SOCS1), a negative regulator of cytokine signaling and suppressor of inflammation related diseases, is also regulated epigenetically.* SOCS1* promoter hypermethylation is one of the best-categorized epigenetic changes in macrophages and hepatocellular carcinoma [52–54]. A recent study provides evidence that loss of* SOCS1* expression inside tumor cells via promoter hypermethylation is strongly associated with overproduction of inflammatory cytokines like TNF-*α* and IL-6. [55].

Inflammatory cytokines such as CXCL1/GRO*α* exert cancer-promoting activities by increasing tumor angiogenesis. CXCL1/GRO*α* decreases the expression of extracellular matrix and plasma protein, fibulin-1D (gene symbol:* FBLN1*) in prostate cancer cells [56].

In highly invasive castration-resistant prostate cancer (CRPC), CXCL1/GRO*α* signaling results in nuclear factor-kappa B (NF-*κ*B) activation that interacts with histone deacetylase 1 (HDAC1) and form gene-silencing complex. This complex represses the expression of fibulin-1D by deacetylation of histones H3 and H4 on the NF-*κ*B-binding site of the fibulin-1D promoter [56].

## 4. The Interplay between Epigenetically Regulated Cytokines and Cancer

Epigenetic changes can alter the pattern of normal gene expression thus resulting in pathological conditions including cancer. Aberrant epigenetic events are common in cancer [57]. These changes typically result in silencing of tumor suppressor genes or activation of tumor inducing genes. The transcriptional silencing usually involves hypermethylation of gene promoters while overexpression is a result of DNA hypomethylation. Various inflammatory cytokines are regulated aberrantly in human cancer via epigenetic mechanisms [58–60].

Although epigenetic regulation, especially aberrant DNA methylation, is considered a common mechanism in tumorigenesis, there are only few studies regarding the role of epigenetic regulations of cytokines in malignancies. It is evident that inflammatory cytokines and chemokines, for example, tumor necrosis factor-alpha (TNF-*α*), IL-1 and IL-6, and interferon gamma (IFN-*γ*), which can be produced by the tumor cells and/or tumor-associated leukocytes and platelets, may add directly to the development of malignancy. Cytokines can also mediate the activities of immune cells in the fight against malignant cells [61].

### 4.1. Chemokines Hypermethylation in Cancer

Human chemokine superfamily is composed of more than 50 small secreted proteins. Recent evidences show that chemokines and their receptors also play a critical role in neoplastic transformations, cancer progression, and angiogenesis, in addition to their role in development and inflammatory responses [62]. CXCL14 also known as BRAK is a member of chemokine family. It acts as a chemoattractant and serves immunity by stimulating trafficking of natural killer cells to the sites of inflammation or malignancy [63]. Several parallel studies show that CXCL14 mRNA and protein are universally expressed in normal tissues but are absent in tumor cell lines and primary tumors [64, 65]. CXCL14 was found to be transcriptionally inactivated by promoter CpG hypermethylation in human prostate cancer,* in vitro* demethylation of the promoter in PC3 cells reexpressed the chemokine [66]. Aberrant methylation of CpG islands in promoter region and the first exon of the gene was associated with its downregulation in gastric cancer [67]. CXCL14 is also known to control colorectal cancer by inhibiting migration and invasion by suppressing NF-*κ*B signaling. Apart from gastric cancer CXCL14 is silenced in colorectal cancer by frequent methylation of the promoter region [68]. Another member of the same family, CXCL12, and its receptor CXCR4 are also associated with tumorigenesis. Interaction between chemokines CXCL12 and CXCR4 promotes cellular adhesion, survival, proliferation, and migration. Its upregulation is reported in skin, lung, pancreas, brain, and breast cancer, while, in pancreatic cancer and melanoma, CXCR4 is downregulated by promoter aberrant methylation [69–71]. Breast cancer cells, with upregulated CXCR4 genes, have been found engrossed to CXCL12 expressing cells in the lymph nodes, liver, and lungs, thus causing metastasis of disconnected tumor cells [72]. Epigenetic mechanisms like demethylation of CXCR4 and hypermethylation of CXCL12 and ESR1 are a characteristic of tumor stage, size, metastasis, and poor overall survival [73]. Knowing the methylation status of both these genes can serve as a biomarker for diagnosis and prognosis in breast cancer [74].

### 4.2. Epigenetic Regulation of Interleukins in Cancer

Many proinflammatory cytokines including interleukins are frequently claimed to be epigenetically regulated in cancers, especially lung cancer [75–77]. DNA methylation at the promoters of IL-1B, IL-6, and IL-8 genes and their consequential downregulation is reported in lung cancer [59]. Among them, IL-1B promoter has the highest methylation status. In addition, IL-23, a member of the IL-6 superfamily that plays a key role in cancer, is also epigenetically modified in malignancies. It promotes inflammatory responses in the defense against pathogenic infection and upregulation of angiogenic factors and MMPs [78]. Recently, interleukin-23 (IL-23) is claimed to be epigenetically regulated in non-small-cell lung cancer (NSCLC) by both histone acetylation and DNA methylation [79]. IL-23A together with its receptor IL-23R mediates inflammatory pathways; the receptor is also regulated by epigenetic mechanisms in lung cancer [80].

Another member of the same family, IL-12 is known to inhibit directly the growth of human lung adenocarcinoma [81]. It holds a powerful antitumor potential, due to activation of combined immune stimulatory and antiangiogenic mechanisms [82–84]. IL-12 facilitates cytotoxic natural killer cells and induces the production of IFN-*γ* from NK and T cells. In addition, IL-12 downregulates the production of the proangiogenic factors VEGF and FGF-2 [85–88].

The IL-12 receptor (R) contains two subunits IL-12Rb1 and IL-12Rb2 [89].* IL12RB2* gene encodes IL-12R chain essential for the IL-12 signal transduction [82, 90]. Epigenetic silencing of* IL12RB2* is a recurrent event in human lung cancers. Aberrant methylation of this gene sounds like a useful forecaster of long-standing result for adenocarcinoma of lung [91].* IL12RB2* methylation is also reported to be a more frequent in the patients suffering from both chronic obstructive pulmonary diseases (COPD) and non-small-cell lung cancer (NSCLC) [92].

### 4.3. Epigenetic Regulation of Interferons in Cancer

Interferons are fighters against viral invaders. IFN-*γ* is a pleiotropic cytokine secreted by type-1 helper (Th1) T cells, cytotoxic T cells, and stimulated natural killer. Production of IFN-*γ* is related to the induction of reaction in T lymphocytes, which contributes to enhancement of an immune response against malignant cells. IFN-*γ* (gene symbol:* IFNG*) stimulates antitumor immune activity by inhibiting cell proliferation and sensitizes tumor cells to apoptosis [93, 94]. Downregulation of* IFNG* mediated by hypermethylation has been observed in lung and cervical cancer [95, 96]. Human papillomavirus (HPV) is now a well-known risk factor involved in the progression of cervical cancer targeting keratinocytes which produces IFN-*κ* [97]. A recent study reported that IFN-*κ* is suppressed in the presence of E6, a HPV protein, signifying the involvement of E6 in IFN-*κ de novo* methylation followed by transcriptional silencing [98].

## 5. Epigenetic Targeting Agents in Cancer Therapeutics

Epigenetic therapy is emerging as an exciting, novel approach to treat a variety of diseases, particularly cancer. This therapy consists of using DNA methylation inhibitors and HADC inhibitors for the reversal of the epigenetic aberrations inside the diseased genome. The reversal of aberrant gene methylation is more considerable to reversal of gene mutations or deletions. Epigenetic drugs, whether demethylating agent or HDAC inhibitor, target aberrantly heterochromatic regions, leading to reactivation of tumor suppressor genes and/or other genes that are vital for the normal cells [99]. There are two classes of DNA methylation inhibitors: nucleoside analogues and nonnucleoside analogues. DNA methylation inhibitors include 5-azacytidine (5-Aza-CR), zebularine, 5-aza-2′-deoxycytidine (5-Aza-CdR), and 5-fluoro-2′ deoxycytidine (5-F-CdR), and so forth. 5-Aza-CR and 5-Aza-CdR have been widely studied for the treatment of hematological diseases. 5-Aza-CR is approved by FDA for the treatment of myelodysplasia in 2004 [100]. HDAC inhibitors include short chain fatty acids, hydroxamic acids, cyclic tetrapeptides and benzamides, each of them possesses different functional groups.

Many histone acetylases and deacetylases have been identified but their specific inhibitors still need to be investigated. These agents inhibit histone deacetylase enzymes, so histones remain acetylated and tailed by changes in cellular processes that were malfunctioning in malignant cells. Unlike chemotherapeutic drugs, demethylating agents do not mark cells for instant death. The cells are left to proliferate and reactivate the aberrantly hypermethylated genes. Inhibition of DNA methylation and consequently reactivation of genes, including apoptotic genes and cell-cycle regulators, finally lead the transformed cell to death and cell-cycle arrest [101]. Many investigators have combined DNA-methylation inhibitors with HDAC inhibitors and have shown synergistic tumour-cell-growth inhibition and gene reexpression [102, 103]. It might be advantageous to combine both drugs for the treatment of solid tumors because treatment with DNA-methylation inhibitors alone in solid tumors is not sufficient [103]. Epigenetic targeting of different misregulated genes inside human malignancies is currently in phases I, II, and III clinical trials, to restore them back.

Considering the great potential of epigenetic therapy, there is a hope that in near future it will be possible to target the aberrant regulation of inflammatory cytokines including interleukins, interferons, and chemokines that are facilitating the malignancies. Complete understanding of these epigenetic modifiers and their specific target cytokine will make development of the most effective therapies possible, not only to treat but also to prevent cancer.

## 6. Conclusion

The literature cited in this review demonstrates that aberrant epigenetic regulation of diverse members of inflammatory cytokines inside different cancers is linked to tumor initiation, endurance, invasion, and progression in one way or the other. Both under- and overexpression of these cytokines are dependent on their epigenetic regulations. Epigenetically regulated cytokines mediate the expression of tumor-associated genes and manipulate their biological role in cancer. Chemokines and interleukins appear to be actively regulated in lung cancer. Targeting the reversal of aberrantly regulated cytokines could be a good potential target for cancer therapy and advanced research in this field could enable us to develop more efficient methods for cancer treatment.
